# Intraoperative burst suppression is associated with postoperative delirium following cardiac surgery: a prospective, observational study

**DOI:** 10.1186/s12871-015-0051-7

**Published:** 2015-04-28

**Authors:** Martin Soehle, Alexander Dittmann, Richard K Ellerkmann, Georg Baumgarten, Christian Putensen, Ulf Guenther

**Affiliations:** 1 Department of Anaesthesiology and Intensive Care Medicine, University of Bonn, Bonn, Germany; 2 Department of Neurology and Psychiatry, LVR-Clinic, Bonn, Germany

**Keywords:** Cardiac surgery, Postoperative delirium, Outcome, Electroencephalogram, Burst suppression, Bispectral Index

## Abstract

**Background:**

Postoperative delirium (POD) occurs frequently after cardiac surgery and is associated with increased morbidity and mortality. We analysed whether perioperative bilateral BIS monitoring may detect abnormalities before the onset of POD in cardiac surgery patients.

**Methods:**

In a prospective observational study, 81 patients undergoing cardiac surgery were included. Bilateral Bispectral Index (BIS)-monitoring was applied during the pre-, intra- and postoperative period, and BIS, EEG Asymmetry (ASYM), and Burst Suppression Ratio (BSR) were recorded. POD was diagnosed according to the Confusion Assessment Method for the Intensive Care Unit, and patients were divided into a delirium and non-delirium group.

**Results:**

POD was detected in 26 patients (32%). A trend towards a lower ASYM was observed in the delirium group as compared to the non-delirium group on the preoperative day (ASYM = 48.2 ± 3.6% versus 50.0 ± 4.7%, mean ± sd, p = 0.087) as well as before induction of anaesthesia, with oral midazolam anxiolysis (median ASYM = 49.5%, IQR [47.4;51.5] versus 50.6%, IQR [49.1;54.2], p = 0.081). Delirious patients remained significantly (p = 0.018) longer in a burst suppression state intraoperatively (107 minutes, IQR [47;170] versus 44 minutes, IQR [11;120]) than non-delirious patients. Receiver operating analysis revealed burst suppression duration (area under the curve = 0.73, p = 0.001) and BSR (AUC = 0.68, p = 0.009) as predictors of POD.

**Conclusions:**

Intraoperative assessment of BSR may identify patients at risk of POD and should be investigated in further studies. So far it remains unknown whether there is a causal relationship or rather an association between intraoperative burst suppression and the development of POD.

**Trial registration:**

clinicaltrials.gov NCT01048775

## Background

Delirium is defined as an acute disturbance of consciousness with a fluctuating course that affects attention, cognition, emotionality, and the sleep-wake cycle [1]. Following cardiac surgery using extracorporeal circulation, postoperative delirium (POD) occurs frequently with a reported incidence ranging between 14 and 51% [2-5]. It is associated with a prolonged stay in the intensive care unit, as well as an increased morbidity and mortality [5-7]. Hence, the success of cardiac surgery is seriously imperiled by the development of POD, and measures to predict and prevent POD are urgently sought.

The electroencephalogram (EEG) has been shown to be affected by POD in terms of an increased delta- and theta- as well as a decreased alpha- and beta-activity [8-10]. The main generators of this delirium-related theta increase have been reported to be localized in the anterior cingulate cortex as well as the right fronto-temporal brain areas [11]. A classic multi-lead EEG, recorded from the full set of scalp electrodes, is impractical in the acute clinical setting of anaesthesia and impossible to be analysed online by the untrained anaesthetist. Processed EEG monitors, such as the Bispectral Index (BIS) monitor, record one or two EEG channels only, and transform the complex EEG information into a few quantitative numbers, [12,13] such as the BIS, the EEG Asymmetry (ASYM) or the Burst Suppression Ratio (BSR). Even though the BIS monitor has been developed to quantify depth of anaesthesia (and not the degree of delirium), it might nevertheless provide useful clinical information to identify patients at risk for POD or even predict POD. With the increased EEG activity in right frontal-temporal brain areas during POD, [11] our hypothesis was that patients with postoperative delirium will have EEG asymmetry not seen in non-delirious patients. This may occur at any time in the perioperative course. POD as well as the occurence of EEG burst supression has been shown independantly to be associated with an increased mortality, [14,15] however the relation between burst suppression and POD has remained unclear. We hypothesized that patients with postoperative delirium will spend more time in burst suppression and exhibit a higher burst suppression rate than nondelirious patients. We therefore performed a prospective study to test these hypotheses.

## Methods

This study has been performed in accordance with the Declaration of Helsinki and has been approved by an appropriate ethics committee (Ethik-Kommission der Medizinischen Fakultät, Rheinische Friedrich-Wilhelms-Universität, Bonn, Germany; Approval No. 056 / 09). With written informed consent, we performed a prospective observational study, which was registered at ClinicalTrials.gov (NCT 01048775). Patients with an age of at least 60 years who were scheduled for elective cardiac surgery were included in the study. Exclusion criteria were pregnancy, emergency or off-pump cardiac surgery.

### BIS monitoring

Bilateral BIS monitoring was applied to the patient’s forehead as recommended by the manufacturer and connected to a BIS VISTA Monitor (version: platform 2.03, application 3.00, hardware revision 3.00). Data were sampled in 5-second intervals for later analysis. Three parameters were analyzed offline: BIS, ASYM and BSR. The BIS quantifies the depth of anaesthesia in a range between 100 (fully awake patient) and 0 (isoelectric EEG) [16]. ASYM denotes the asymmetry in the EEG total power (within the frequency range from 0–30 Hz) comparing the left and right hemisphere, according to the formula [17]:

$$ ASYM = \left( total\  power\  left\ /\  total\  power\  left + right\right)\ *\  100\ \% $$

Hence, an ASYM of 50% represents equal total power in both hemispheres, whereas an ASYM < 50% indicates less total power in the left than the right hemisphere. The Burst Suppression Ratio (BSR) is defined as the percentage of epochs in the previous 63 seconds in which the EEG signal is considered suppressed [18]. Suppression is recognized as those periods longer than 0.5 seconds, during which the EEG voltage does not exceed approximately ± 5 μV [18,19].

In the preoperative period, the bilateral BIS was monitored in a calm surrounding on the afternoon prior to surgery. To do so, patients were asked to lie down on their bed, to close their eyes, and to relax during the BIS recording, which was performed by the same investigator (AD) over a period of 20 minutes in a standardized manner. On the following day, the bilateral BIS was recorded from entering the induction room during the entire surgery (intraoperative period). The EEG in the period from induction until onset of cardiopulmonary bypass (CPB) was analysed separately since this epoch was considered to be unaffected by any inflammatory process triggered by CPB. Bilateral BIS monitoring was continued during the intensive care unit (ICU) stay (postoperative period) until discharge. The same BIS sensor was used for the entire period, unless an insufficient adhesion required a sensor replacement. During cardiac surgery, anaesthetists were blinded to the BIS and asked to perform anaesthesia according to clinical routine.

### Anaesthesia

Oral midazolam (7.5 mg) was administered one hour before induction of anaesthesia. Anaesthesia was induced with etomidate (~0.2 mg/kg b.w.), sufentanil (10 μg/kg/h until a total dose of ~ 0.5 μg/kg b.w. was reached) and cis-atracurium (0.15 mg/kg b.w.). The trachea was intubated and anaesthesia was maintained with isoflurane (~0.7 MAC), sufentanil (~1 μg/kg/h) and cis-atracurium. Patients were transferred from the induction room to the adjacent operating room at the discretion of the attending anaesthesiologist. During CPB, isoflurane was directly applied to the extracorporeal oxygenator and monitored using the gas analyzer of the anaesthesia ventilator. Following surgery, all patients were transferred to ICU, where patients were sedated with continuous propofol and sufentil according to our institution’s standard operating procedure. Sedation was stopped as soon as the patients were in a stable condition.

### Monitoring for delirium and outcome

Postoperatively, patients were monitored for the occurrence of POD using the flowsheet version of the Confusion Assessment Method for Intensive Care Units (CAM-ICU-Flowsheet), which is explained in more detail elsewhere [20,21]. The CAM-ICU was performed once every day by the same investigator (AD). According to CAM-ICU criteria, [20,21] patients were rated as having POD if they were CAM-ICU positive for at least a single examination. Depending on their Richmond Agitation-Sedation Score (RASS), [22] delirious patients were subgrouped as either hypoactive (RASS ≤ 0) or hyperactive (RASS > 0). Patients that alternated between positive and negative RAS scores were grouped as having a mixed delirium [23]. Activity of Daily Living was assessed on the day prior to surgery as well as 6 months after: To do so, a questionnaire was completed in which the “Alzheimer’s Disease Cooperative Study Mild Cognitive Impairment Activities of Daily Living Scale” (ADCS-MCI-ADL) was determined, [24] which ranges from 0 (completely depending on support) to 57 (totally independent). Mortality was assessed at 6 months after surgery.

### Statistics

Patients were divided into a group with and without delirium, and data were averaged within groups. Data are shown as mean ± standard deviation in case of normal distribution or otherwise as median and interquartile range. Groups were compared by *t*-test or Mann–Whitney rank sum test, respectively. Receiver operating characteristic (ROC) analysis was performed to investigate the sensitivity, specificity and predictive value of certain parameters to predict POD. ROC analysis and statistical tests were performed using SigmaPlot-Software (version 12.3, Systat Software Gmbh, Erkrath, Germany). Statistical significance was assumed at p < 0.05.

## Results

### Epidemiology

Eighty-seven patients (n = 87) were included into the study, four of whom died during their intensive care stay (perioperative mortality rate = 4.6%). These were excluded since they could not be screened for the occurrence of POD. Another two patients were excluded: One withdrew consent, the other was unintentionally not intraoperatively monitored. Therefore, eighty-one patients (n = 81) remained for analysis:

Patients consisted of 24 women and 57 men with a mean age of 72.9 ± 6.2 years, a mean height of 171.3 ± 8.5 cm and a median weight of 76 [70; 88] kg (Table 1). Coronary artery bypass grafting (CABG) was performed in 44, valve replacement or reconstruction in 22, and a combined CABG/valve-surgery in 15 patients. Surgery lasted for 5.4 ± 1.2 hours, of which 2.2 ± 0.6 hours were spent in CPB and 1.5 ± 0.5 hours in aortic clamping.

**Table 1:** **Patient characteristics**

			Patients		p-value
Group		All	With delirium	Without delirium	
Number		81	26	55	
Age	[years]	72.9 ± 6.2	74.5 ± 6.5	72.1 ± 5.9	0.101
Gender	[m/f]	57/24	16/10	41/14	0.299
Height	[cm]	171.3 ± 8.5	169.6 ± 8.2	172.2 ± 8.6	0.212
Weight	[kg]	76.0 [70.0; 88.0]	74.0 [69.3; 78.3]	80.0 [70.0; 92.0]	0.083
Surgery:					
	CABG	44	13	31	0.638
	Valve	22	8	14	0.605
	Misc	15	5	10	1.000

Data are displayed as mean ± std dev in case of normal distribution or otherwise as median and interquartile range. CABG = coronary artery bypass grafting. Statistic analysis revealed no significant differences in the above mentioned parameters between patients with and without delirium

### Postoperative delirium

**Figure 1:**
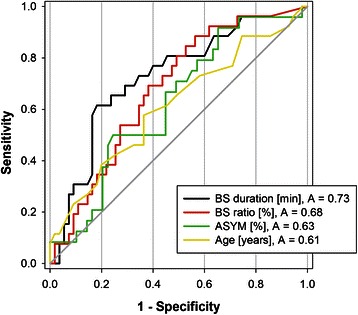
Receiver operating characteristic (ROC) analysis of four parameters to predict postoperative delirium. Burst suppression (BS) was assessed on the right side during the period from induction of anaesthesia until the onset of cardiopulmonary bypass. The area under the curve (A) for the parameters BS duration and BS ratio was significantly (p = 0.001 and 0.009 respectively) different from 0.5, whereas the area under the curve for age and ASYM was not. ASYM = EEG asymmetry.

Twenty-six patients (32%) developed POD. Patients with and without POD did not differ statistically with respect to age, gender, height and type of surgery (Table 1). However patients with delirium tended to have a (non significantly) higher age (74.5 ± 6.5 years, p = 0.10) as well as a (non significantly) lower body weight (median of 74 [69; 78] kg, p = 0.08) as compared to non-delirious patients (age = 72.1 ± 5.9 years, weight = 80 [70; 92] kg, Table 1). No statistical difference was observed between patients with or without delirium with respect to comorbidities, preoperative serum electrolytes and intraoperative parameters, such as arterial blood pressure, body temperature or the amount of anaesthetic drugs administered (Tables 2 and 3). ROC analysis for age yielded an area under the curve of 0.61, which was statistically not significant (p = 0.10, Figure 1, Table 4). Hence, age was not a predictor of POD in our study.

**Table 2:** **Comorbidity and plasma electrolyte concentrations in comparison between delirious and non-delirious patients**

		Patients with delirium (n = 26)	Patients without delirium (n = 55)	p-value
**Comorbidity**				
Congestive heart failure		9	24	0.48
Myocardial infarction		6	18	0.44
Diabetes mellitus		7	15	1.00
COPD		5	8	0.75
Peripheral vascular disease		2	5	1.00
Cerebrovascular disease		3	4	0.20
**Preoperative plasma electrolyte concentrations**				
Sodium	[mmol/l]	140 [137.8;142.3]	140 [139;141]	0.83
Potassium	[mmol/l]	3.7 [3.6; 4.1]	3.8 [3.5;4.1]	0.96

The number of patients with certain comorbities is shown in the upper part. COPD = chronic obstructive pulmonary disease. Electrolyte concentrations are expressed as medians and interquartile range. Groups did not differ significantly with respect to the above shown parameters.

**Table 3:** **Intraoperative parameters**

		Patients		p-value
		With delirium	Without delirium	
Duration of surgery	[min]	321 ± 69	326 ± 74	0.768
CPB time	[min]	131 ± 33	129 ± 35	0.786
**Anaesthetic agents**				
Sufentanil (total dose)	[μg]	406 ± 97	417 ± 106	0.679
Isoflurane (average)	[etVol %]	0.7 ± 0.1	0.7 ± 0.1	0.679
Average **arterial blood pressure**				
Intraoperatively	[mmHg]	87.8 ± 7.0	86.6 ± 5.6	0.464
During CPB	[mmHg]	65.1 ± 5.4	62.7 ± 6.1	0.125
Average **body temperature**	[°C]	35.6 ± 0.4	35.6 ± 0.4	0.515

Data are displayed as mean ± std dev. CPB = cardiopulmonary bypass. No significant differences between the groups were observed for any of the above mentioned parameters.

**Table 4:** **Receiver operator characteristic (ROC) analysis regarding the ability of four parameters to predictor postoperative delirium**

Parameter	Area under the curve	p-value	Cut-off	Sensitivity	Specificity	Positive predictive value	Negative predictive value
Burst suppression duration	0.73	0.001	47 min.	0.58	0.84	0.63	0.81
Burst suppression ratio	0.68	0.009	5%	0.23	0.89	0.50	0.71
ASYM	0.63	0.08					
Age	0.61	0.10					

Burst suppression values for duration and ratio were obtained from the right forehead during the period from induction of anaesthesia until the onset of cardiopulmonary bypass. No cut-off data or predictive values were calculated for the parameters age and ASYM, since the area under their curve was non-significantly (p > 0.05) different from 0.5 (diagonal line).

The hypoactive subtype was observed as the dominant type of delirium, which occurred in 20 patients (77%). The mixed and the hyperactive subtype were diagnosed in 5 and 1 patient, respectively. Patients with delirium spent almost double the time in ICU (median = 81 [23; 141] hours) as compared to non-delirious patients (median = 42 [20; 90] hours, p = 0.033).

The intraoperative BIS did not differ between patients who later developed POD (BIS = 44.6 ± 5.5) and those who never developed POD (BIS =45.1 ± 7.7).

Preoperative and 6-month ADCS-MCI-ADL scores did not differ between surviving patients with postoperative delirium and those who were not delirious. At 6-months, a significantly (p = 0.03) higher mortality was observed in the delirium group (11.5%) as compared with the non-delirium group (0%).

### EEG asymmetry

**Figure 2:**
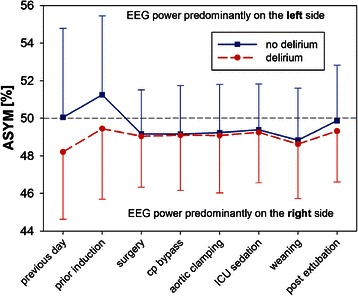
The time course of the EEG-asymmetry (ASYM) in comparison between delirious and non-delirious patients. An ASYM of less or more than 50% indicates more EEG power on the right or left hemisphere, respectively. Cp bypass = cardiopulmonary bypass. Data = mean ± std dev.

ASYM increased (non-significantly) from 49.4 ± 4.5% on the previous day to 50.7 ± 4.1% prior to induction. Apart from this initial rise, ASYM was approximately 49 ± 3% for most of the remaining time, indicating a slightly higher total EEG power over the right hemisphere. Patients who later on developed POD showed a nonsignificant trend towards a lower ASYM on the previous day (48.2 ± 3.6% versus 50.0 ± 4.7%, p = 0.087) and prior induction (49.5% [47.4;51.5] versus 50.6% [49.1;54.2], p = 0.081) as compared to patients who never developed POD (Figure 2). At other time points, we observed no ASYM difference between the groups. ASYM was not a predictor of POD, since ROC analysis indicated an AUC of 0.63, which was not significantly different from 0.5 (p = 0.08, Figure 1, Table 4)

### EEG burst suppression

**Figure 3:**
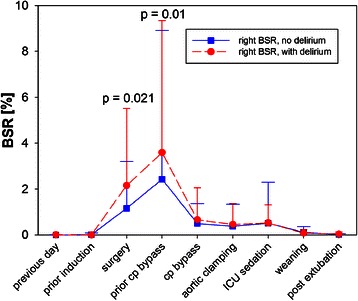
The Burst Suppression Ratio (BSR) as observed on the right side during the pre-, intra- and postoperative period. Data obtained in delirious or non-delirious patients are illustrated in red and blue, respectively. Cp bypass = cardiopulmonary bypass. Data = mean ± std dev.

During the ICU stay, we observed no difference in BSR between patients who developed delirium and those who did not. However during surgery, a significantly (p = 0.028) higher BSR was observed in patients who subsequently developed POD (BSR = 1.24%, IQR [0.30;3.34]) as compared to those who did not (BSR = 0.44%, IQR [0.06;2.00]). A significant difference in BSR between the groups was also observed for the time period between intubation and onset of cardiopulmonary bypass (Table 5). This phenomenon occurred on the left as well as the right side (Figure 3). Although burst suppression was observed during surgery, the BSR itself was low (<10%).

**Table 5:** **Burst Suppression Ratio during surgery**

	Patients		
Group	With delirium	Without delirium	p-value
**Averaged over the entire period of surgery**			
Left BSR	1.4% [0.3;5.3]	0.5% [0.1;2.3]	0.032
Right BSR	0.7% [0.2;2.9]	0.4% [0.04;1.3]	0.021
**Averaged over the period from intubation to onset of cardiopulmonary bypass**			
Left BSR	1.8% [0.3;4.9]	0.5% [0.05;2.5]	0.035
Right BSR	1.3% [0.4;4.5]	0.3% [0.02;2.7]	0.010

All data are shown as median and interquartile range.

During surgery, patients who later on developed delirium spent significantly (p = 0.018) more time (107 minutes, IQR [47;170]) in a state of burst suppression than non-delirious patients (44 minutes, IQR [11;120], Table 6). ROC analysis identified BS duration as well as BS ratio as significant predictors of POD with an area under the curve of 0.73 and 0.68, respectively (Figure 1, Table 4). For cut-off values of 47 minutes BS duration and 5% BS ratio, sensitivity, specificity and predictive value are shown in Table 4.

**Table 6:** **Time spent in a state of burst suppression, i.e. a BSR > 0%**

	Patients		
Group	With delirium	Without delirium	p-value
**Duration of burst suppression as obtained during the entire period of surgery**			
Left side	131 min [50;183]	48 min [13;127]	0.034
Right side	85 min [46;142]	35 min [7;89]	0.009
**Duration of burst suppression from intubation to onset of cardiopulmonary bypass**			
Left side	59 min [17;77]	20 min [3;58]	0.008
Right side	53 min [18;77]	13 min [2;37]	0.001

All data are shown as median and interquartile range.

## Discussion

### Asymmetry

In our study, we applied bilateral BIS monitoring to investigate EEG asymmetry in the perioperative period of cardiac surgery. Contrary to the reported EEG lateralization during POD [11], patients with delirium did not show an altered asymmetry postoperatively in our study. Presumably, such lateralization phenomena might only be detected applying dedicated techniques such as Low Resolution Electromagnetic Tomography [25] or intracranial EEG recordings, but not using a single or dual channel EEG analyzer such as the BIS monitor, which analyses superficial and local electrical activity percutaneously.

However, during the preoperative period (that is, on the previous day as well as prior to induction), we observed a trend towards a lower ASYM (more EEG power on the right side) in patients that later on developed POD. This tendency has not been reported before and requires further investigation. So far, we have no explanation why this difference in ASYM between the delirious and the non-delirious group vanishes as soon as anaesthesia is induced. In addition, we observed an increase in ASYM from the previous day to the period prior induction, which might be related to the premedication (midazolam) given one hour before induction.

### Burst suppression

In our study, patients who later developed POD, exhibited a significantly higher BSR during surgery and remained significantly longer in a state of BSR > 0 as compared to patients who did not develop POD. A possible explanation would be that “excessive” anaesthetic depth, as indicated by the occurrence of burst suppression, might have contributed to the development of POD and an increase in long-term mortality in our study. In fact, high doses of anaesthetics induce a burst suppression EEG, [26-28] and the cumulative duration of “deep” anaesthesia (specifically a BIS < 45) has been shown to be associated with an increased long-term mortality [29-32]. In addition, Watson et al. reported a two-fold 6-month mortality in critically ill patients with a BSR > 0 as compared to patients without burst suppression [15]. However, the anaesthetic depth in our study (median BIS = 41.3 and 44.2 on the left and right side, respectively) was within the recommended range (BIS between 40 and 60) [33] and comparable with other studies of cardiac surgery: For instance, Kertai et al. reported an average intraoperative BIS of 40 ± 7.2 in 460 patients [29]. Moreover, the BIS during surgery did not differ in our study between patients who eventually developed POD and those who did not.

High doses of anaesthetics are associated with a high BSR, and above a BSR threshold of 40%, the BIS is directly and linearly related to the BSR according to the formula BIS = 50 – BSR/2 [26]. In contrast, the BSR values in our study were low (<10%) and in a range, where the BIS remains unaffected. Therefore, it is unlikely that excessive anaesthetic depth was the cause for the low BSR values observed in our study. A mean intraoperative BSR of 7.1 and 8.8% was observed by Radtke et al. [34] during non-cardiac surgery in a BIS guided and a BIS blinded group, respectively, indicating that low BSR values occur during both cardiac and non-cardiac surgery. In fact, higher BSR values (>10%) were observed by Besch et al. in 8.8% of patients undergoing noncardiac surgery [35]. They identified an age > 60 years, history of coronary artery disease and male gender as independent factors associated with a BSR > 10%, which are characteristics also present in the majority of our patients.

In general, an EEG with a high BSR indicates coma due to excessive anaesthetic depth [26] or other etiologies (hypoxia, intoxication, hypothermia, encephalopathy) [36]. However, the pathophysiology and clinical impact of a “low BSR EEG” remains unknown [35]. We speculate that it may indicate an increased sensitivity of the brain to the inflammatory response triggered by surgery. Alternatively, the occurrence of burst suppression at typical anaesthetic doses may identify patients with unusual anaesthetic sensitivity, in whom a “normal” anaesthetic dose is in fact a relative overdose [5]. This hypothesis is supported by Whitlock et al., who identified low average anaesthetic dose as an independent predictor of delirium [5]. Hence studies, which aim at reducing a low BSR EEG are required.

### Delirium

The incidence of POD in our study (32%) is within the reported range from 14 to 51% [3-5]. Patients who developed POD experienced a higher long-term mortality than non-delirious patients in our study, which confirms earlier reports [14,37,38].

### Limitations

One limitation of our study is due to its design as an observational study. It remains unknown whether an intervention would have been effective to reduce or avoid burst suppression during surgery, and whether this would have reduced the incidence of delirium. Another limitation is the exclusion of patients under 60 years of age. It remains unknown whether our results could be extended to younger patients, especially since an age > 60 years has been identified by Besch et al. [35] as a risk factor for the occurrence of a burst suppression EEG during anaesthesia.

## Conclusions

The intraoperative assessment of the Burst Suppression Ratio may help to identify patients at risk for POD following cardiac surgery. So far it remains unknown, whether there is a causal relation or rather an association between BSR and the development of POD. Hence, further studies are required to clarify this question. We deem excessive depth of anaesthesia unlikely to be the underlying reason for the occurrence of burst suppression in our study, since the BIS was within the recommended range and the observed BSR was low. However, the clinical significance of such low BSR values is unknown and requires further investigations.

## Abbreviations

ADCS-MCI-ADL: Alzheimer’s Disease Cooperative Study Mild Cognitive Impairment Activities of Daily Living
ADL: Activity of daily living
ASYM: Asymmetry of the electroencephalogram
BIS: Bispectral Index ®
BSR: Burst suppression ratio
CABG: Coronary artery bypass grafting
CAM-ICU: Confusion assessment method for intensive care units
CPB: Cardiopulmonary bypass
EEG: Electroencephalogram
ICU: Intensive care unit
IQR: Interquartile range
MAC: Minimal alveolar concentration
POD: Postoperative delirium
ROC: Receiver operating characteristic
